# Treatment With Hydrolyzed Diet Supplemented With Prebiotics and Glycosaminoglycans Alters Lipid Metabolism in Canine Inflammatory Bowel Disease

**DOI:** 10.3389/fvets.2020.00451

**Published:** 2020-07-30

**Authors:** Yoko M. Ambrosini, Sebastian Neuber, Dana Borcherding, Yeon-Jung Seo, Sergi Segarra, Barbara Glanemann, Oliver A. Garden, Udo Müller, M. Gordian Adam, Viet Dang, David Borts, Todd Atherly, Auriel A. Willette, Albert Jergens, Jonathan P. Mochel, Karin Allenspach

**Affiliations:** ^1^Department of Biomedical Sciences, College of Veterinary Medicine, Iowa State University, Ames, IA, United States; ^2^Biocrates Life Sciences AG, Innsbruck, Austria; ^3^R&D Bioiberica S.A.U., Barcelona, Spain; ^4^Department of Clinical Studies and Advanced Medicine, University of Pennsylvania College of Veterinary Medicine, Philadelphia, PA, United States; ^5^Department of Veterinary Diagnostic and Production Animal Medicine, College of Veterinary Medicine, Iowa State University, Ames, IA, United States; ^6^Department of Veterinary Clinical Sciences, College of Veterinary Medicine, Iowa State University, Ames, IA, United States; ^7^Department of Food Science and Human Nutrition, College of Human Sciences, Iowa State University, Ames, IA, United States

**Keywords:** metabolomics (OMICS), inflammatory bowel disease, dogs, serum, partial least-squares-discriminant analysis

## Abstract

Canine inflammatory bowel disease (IBD) is a chronic, immunologically mediated intestinal disorder, resulting from the complex interaction of genetic, environmental and immune factors. Hydrolyzed diets are used in dogs with food-responsive diarrhea (FRD) to reduce adverse responses to immunostimulatory proteins. Prebiotics (PRBs) and glycosaminoglycans (GAGs) have previously been demonstrated to show anti-inflammatory activity in the intestinal mucosa. Notably, hydrolyzed diets combined with the administration of PRBs and GAGs offer a promising approach for the treatment of canine IBD. Our aim was to investigate the effects of hydrolyzed diet and GAG+PRB co-treatment on the serum metabolomic profile of IBD dogs. Dogs with IBD randomly received either hydrolyzed diet supplemented with GAGs and PRBs (treatment 1) or hydrolyzed diet alone (treatment 2) for 10 weeks. A targeted metabolomics approach using mass spectrometry was performed to quantify changes in the serum metabolome before and after treatment and between treatment 1 and 2. Principal component analysis (PCA), partial least squares-discriminant analysis (PLS-DA), hierarchical cluster analysis (HCA) and univariate statistics were used to identify differences between the treatment groups. PCA, PLS-DA, and HCA showed a clear clustering of IBD dogs before and after hydrolyzed diet, indicating that the treatment impacted the serum metabolome. Univariate analysis revealed that most of the altered metabolites were involved in lipid metabolism. The most impacted lipid classes were components of cell membranes, including glycerophospholipids, sphingolipids, and di- and triglycerides. In addition, changes in serum metabolites after GAG+PRB co-treatment suggested a possible additional beneficial effect on the lipid metabolism in IBD dogs. In conclusion, the present study showed a significant increase in metabolites that protect gut cell membrane integrity in response to hydrolyzed diet alone or in combination with GAG+PRB co-treatment. Administration of such treatment over 70 days improved selected serum biomarkers of canine IBD, possibly indicating improved intestinal membrane integrity.

## Introduction

Canine inflammatory bowel disease (IBD) is one of the most common chronic gastrointestinal (GI) diseases in dogs (1, 2). It is caused by an interplay of genetic susceptibility, intestinal dysbiosis, diet, and other environmental factors, similar to human IBD (3, 4). Exclusion diets with a single protein and formulation varieties (3) are used to treat such food-responsive enteropathies, including dietary hypersensitivity or food responsive diarrhea (FRD). Several hydrolyzed diets have been developed for the treatment of Crohn's disease in humans (4, 5) and dogs (3). These diets are considered hypoallergenic because the hydrolysis process disrupts protein structures to limit existing allergens and allergenic epitopes, thereby making the diets unlikely to stimulate the immune system (6, 7). Hydrolyzed diets are clinically highly effective for the long-term treatment of both FRE and IBD (8). However, it is not known how such diets impact metabolic processes that coincide with these beneficial effects, which requires the study of serum metabolite profiles.

Chondroitin sulfate (CS), a natural glycosaminoglycan (GAG) found in the extracellular matrix, has been shown to inhibit nuclear factor kappa-light-chain-enhancer of activated B cells (NF-κB) activity (9), which is significantly increased in various chronic inflammatory processes including canine IBD (10). Oral administration of prebiotics (PRBs) has been shown to promote the growth of beneficial gut microbiota (11). Therefore, we hypothesized that oral administration of GAGs and PRBs reduces intestinal inflammation and further benefits dogs with IBD during treatment with hydrolyzed diets. A previous study demonstrated that concomitant treatment with GAGs and PRBs and a hydrolyzed diet was safe in IBD dogs over 180 days, and suggested improvements in selected serum biomarkers, such as cholesterol and paraoxonase-1 (12). Recently, a synergistic effect of PRBs and hydrolyzed diet has been reported in rats, in which goblet cell populations were rapidly restored and malnutrition-induced mucosal atrophy was reduced (13).

Metabolomic analysis in medical research has recently become a promising method for investigating mechanisms that cause chronic diseases, including IBD (14–17). Metabolite profiles can be used not only for prognostic purposes, but also to identify early biomarkers before enteropathic abnormalities are evident (18, 19). Furthermore, metabolomics can be used to investigate mechanistic relationships between certain metabolites and the influence of diets (20, 21). For example, lipid metabolism in humans and rodent models changes in response to the presence of IBD (22–24).

In veterinary medicine, only a few studies have utilized serum metabolomics analysis in clinical patients (25, 26). To our knowledge, only two studies have been reported in dogs with IBD (27, 28). These studies have provided some new insights into metabolomic differences in IBD dogs vs. healthy dogs and IBD dogs before and after treatment. The aim of the present study was to further characterize serum metabolite profiles before and after a hydrolyzed diet therapy in IBD dogs. We also investigated the effect of PRBs and GAGs on metabolite profiles of IBD dogs compared to standard treatment (hydrolyzed diet only) plus placebo therapy.

## Materials and Methods

### Animals

Client-owned dogs with IBD were enrolled in a prospective, randomized, double-blind, placebo-controlled study. All animal owners gave written informed consent. All procedures involving animals were reviewed and approved by the Animal Welfare and Ethical Review Board of the Royal Veterinary College (P302A3B70) and the UK Home Office under the Animals (Scientific Procedures) Act 1986 (PPL 70/7393).

All dogs were diagnosed with IBD based on clinical exclusion diagnosis (29) including stool examination, complete blood count, chemistry profile, urinalysis, serum trypsin-like immunoreactivity, serum canine pancreatic lipase immunoreactivity, serum cobalamin (i.e., B12), and folate concentrations. Furthermore, dogs were analyzed by two-dimensional abdominal ultrasound and endoscopic biopsies of the duodenum, ileum, and colon. Based on the guidelines of the World Small Animal Veterinary Association (WSAVA) International Gastrointestinal Standardization Group (30, 31), dogs showed predominantly lymphoplasmacytic infiltration. The response to dietary treatment was assessed with a commercial hydrolyzed diet: Purina Veterinary Diet HA Hypoallergenic Canine Formula (Purina HA; Nestlé Purina Petcare); details of its nutritional components are shown in Table S1. The dogs presenting for a work-up for chronic diarrhea were discharged from the hospital after all diagnostic procedures. The dog owners were instructed to exclusively feed Purina HA until the biopsy results were available. They were contacted ~7 days post discharge to assess the response of their dogs to the diet. If the dogs responded positively to dietary exclusion with improvement in clinical signs such as having a formed stool or less diarrhea, those IBD dogs were included in the study.

### Study Design

Dogs with confirmed IBD and with a positive response to the dietary exclusion were randomized by means of a computer-generated schedule into one of the two treatment groups: GAG+PRB supplement (treatment 1) and placebo (treatment 2) at visit 2. All dogs were switched to a Purina HA hydrolyzed diet since visit 1, which was maintained throughout the study period. Dogs in the GAG+PRB supplement group (treatment 1) received an oral daily dose of 10 mg CS, 215 mg alpha-glucan butyrogenic resistant starch, 26 mg beta-glucans and mannan-oligosaccharides per kg of body weight. Dogs in the placebo group (treatment 2) received a placebo powder, which only contained excipients and flavorings, orally once a day.

**Figure 1:**
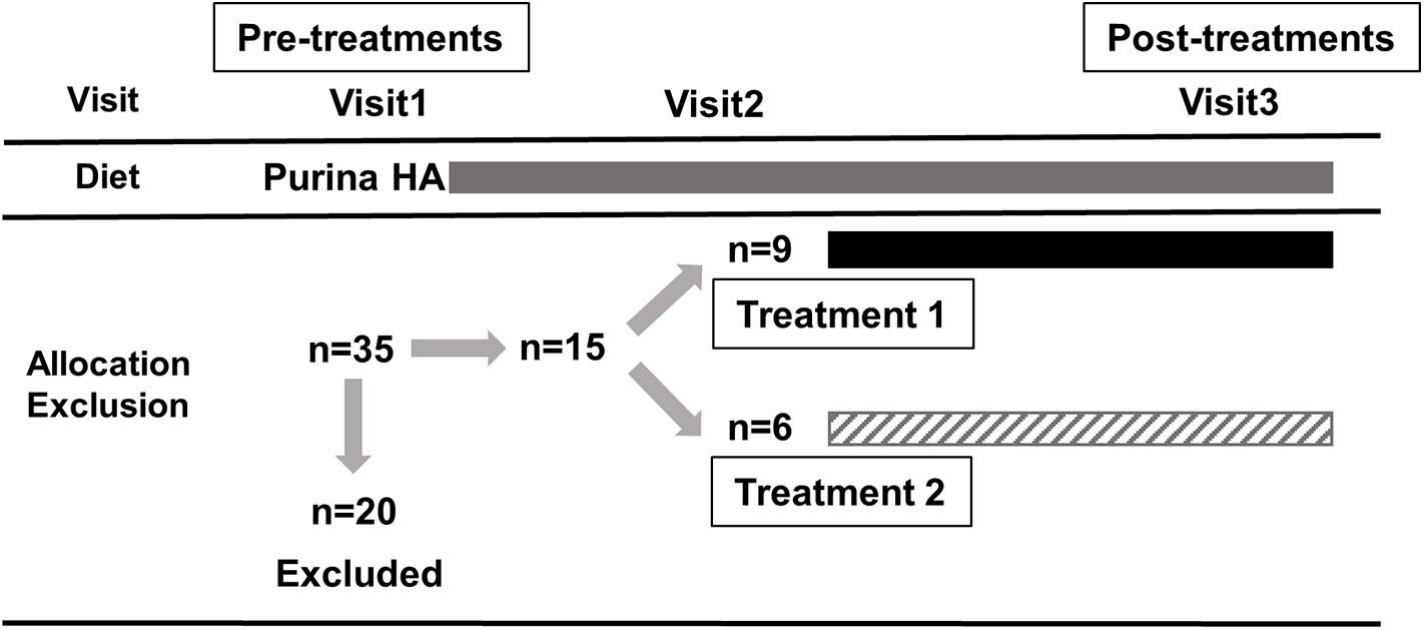
Summary of the trial design and inclusion of dogs. A total of 35 dogs were enrolled and 20 dogs had to be excluded for various reasons, including unscheduled visits, non-compliance with the dosage, progressive weight loss and diarrhea, receiving prior treatment with steroids, cyclosporine, and/or antibiotics. Each box represents the period in which the dogs received Purina HA diet (gray), treatment 1 (hydrolyzed diet supplemented with PRB+GAG; black) and treatment 2 (hydrolyzed diet only; upward diagonal). Blood samples for metabolomics analysis were collected during the initial physical examination (pre-treatment, visit 1, day 0), during a subsequent visit (visit 2, day 14 ± 2), and after the treatment schedule (post-treatment, visit 3, day 70 ± 2).

Blood samples were collected during the initial physical examination (pre-treatment, visit 1, day 0), during a subsequent visit (visit 2, day 14 ± 2), and after the treatment schedule was completed (post-treatment, visit 3, day 70 ± 2). Serum was obtained by centrifugation, frozen within 30 min of collection and then immediately stored at−80°C until analysis. Both the owners and the evaluators of the dogs, including clinicians and those who analyzed samples and tissues, were blinded to the assignment of the treatment groups. The study nursing staff acted as dispensers and were not blinded. None of the IBD dogs had to be unblinded during the course of the study. The trial design and inclusion of dogs are summarized in Figure 1.

Ultimately, a total of 15 dogs were included in this study (*n* = 9 for treatment 1, *n* = 6 for treatment 2). At the time of registration (visit 1), there were 35 dogs, 20 of which were excluded during the study (visit 2) for various reasons, including unscheduled visits, non-compliance with dosage, progressive weight loss and diarrhea, receiving prior treatment with steroids, cyclosporine, and/or antibiotics. The characteristics of included dogs (i.e., breeds, sex, age, and body weight) are summarized in Table 1. The study population consisted of 10 male and 5 female dogs (intact and neutered) of different breeds and crosses aged between 14 and 131 months. For each visit, the owners provided data sheets showing the time and amount of feeding. None of the dogs included in the study had problems eating the recommended daily amount of food. All dogs responded quickly and sustained to dietary treatment for 10 weeks. All dogs remained with their owners at the end of the study and were then subjected to normal veterinary and husbandry practices. Details of the clinical data of the dogs will be published elsewhere in the near future. In short, histopathological evaluations showed a significant decrease in the modified WSAVA histological score (31) after 10 weeks of treatment for both groups, but was not statistically significant between treatment groups.

**Table 1:** Characteristics of the dogs included in the study at the time of enrollment.

	**Treatment 1** **(hydrolyzed diet supplemented with PRBs and GAGs)**	**Treatment 2** **(hydrolyzed diet only)**
Breed	Lurcher Cross Breed Staffordshire Bull Terrier Coker Spaniel Cockapoo Border Collie Labrador Retriever (3)	German Shepherd (2) Boxer Border Collie Collie Cross Lhasa Apso
Sex	Female (0) Neutered female (4) Male (2) Neutered male (3)	Female (1) Neutered female (0) Male (4) Neutered male (1)
Age (months)	54.5 (14–131)	36 (19–80)

*Numerical data are expressed as median (range)*.

### Chemicals

Ammonium acetate (Optima LC-MS), formic acid (mass spectrometry grade), pyridine (99+%, extra pure), isopropanol, acetonitrile, methanol, and water were purchased from Fisher-Scientific (Fair Lawn, NJ 07410). Ethanol was purchased from Merck (Billerica, MA 01821). All solvents were of LC-MS analytical grade or higher purity. Phosphate-buffered saline (PBS 1x, sterile ultra-pure grade) was obtained from VWR (Solon, OH 44139). Phenyl isothiocyanate (PITC, for protein sequencing) was obtained from Sigma-Aldrich (St. Louis, MO 63103).

### Metabolite Extraction

Sample preparation was performed using the AbsoluteIDQ® p400 HR kit (Biocrates Life Sciences AG, Innsbruck, Austria) in accordance with the user manual. In brief, after the addition of 10 μL of the supplied internal standard solution to each well on a filter spot of the 96-well extraction plate, well-pipetting was done using 10 μL of each serum sample, quality control samples, blank, or zero sample (PBS). The plate was then dried under a gentle stream of nitrogen for 30 min using a pressure manifold. The samples were derivatized with 5% PITC in ethanol:water:pyridine (1:1:1 v/v/v) for the amino acids and biogenic amines, and subsequently dried for 60 min under nitrogen. Metabolites and internal standards were then extracted with 300 μL methanol containing 5 mM ammonium acetate by shaking for 30 min at 450 rpm, and eluted by a gentle nitrogen stream. One-half of the eluate was transferred to a new 96-well plate and diluted with water (50:50 v/v) for the liquid chromatography-mass spectrometry (LC-MS) analysis. The second-half of the eluate was diluted with 250 μL of the running solvent for flow injection analysis-mass spectrometry (FIA-MS). Both LC and FIA plates were securely covered with silicon mats and shook for 5 min at 500 rpm prior to analysis.

### Quantification of Metabolites

Metabolites were measured with a targeted metabolomics approach using a Dionex Ultimate 3000 ultra-high-performance liquid chromatography (UHPLC) coupled to a Thermo Scientific Q Exactive Focus Orbitrap mass spectrometer. This method allowed for the quantification of up to 408 endogenous metabolites across 11 different metabolite classes. A total of 21 amino acids and 21 biogenic amines were quantitatively analyzed by UHPLC-electrospray ionization tandem mass spectrometry. The chromatographic separation was based on a reserved-phase liquid chromatography using 0.1% formic acid in water and 0.1% formic acid in acetonitrile as mobile phase A and B, respectively.

The needle wash solvent was consisted of a mixture of acetonitrile, methanol, isopropanol, and water (1:1:1:1 v/v/v/v). The injection volume was set as 5 μL and the flow rate was set as 0.8 mL/min. The remaining 366 metabolites, including 55 acylcarnitines, 172 phosphatidylcholines (PCs), 24 lysophosphatidylcholines (LPCs), 18 diglycerides (DGs), 42 triglycerides (TGs), 31 sphingomyelins (SMs), 9 ceramides, 14 cholesteryl esters, and 1 sum of hexoses were analyzed by FIA-MS, using a one-point internal standard calibration. In terms of quantification, the lipids and a subset of acylcarnitines are semi-quantitative in nature because of (i) the lack of commercially available specific internal standards and (ii) the impossibility to verification accuracy beyond arbitrary luminescence units. Thus, the total concentrations of possible isobars and structural isomers were represented in the present kit. We utilized the running solvent provided by the kit for the FIA-MS analysis and the solvent was used with an injection volume set to 20 μL. Separation gradients and mass detection were performed as recommended by the manufacturer's instructions.

### Data Pre-processing

A total of 53 serum samples from 15 dogs (one to three samples per dog before and after treatment) were included in this study and means were calculated for each dog at each timepoint. The data were then cleaned using a modified 80% rule. Briefly, metabolites that were not present in at least 80% of the samples in at least one group (i.e., pre-treatment 1, post-treatment 1, pre-treatment 2, or post-treatment 2) were discarded; the remaining 284 metabolites included in the present work are listed in Table S2. Remaining values below the limit of detection (LOD) in the data set were replaced applying a logspline imputation method with values between LOD and LOD/2 method (32) (R version 3.4.1, package logspline). To meet the assumptions of statistical tests, the data were additionally log2-transformed (33).

### Multivariate Statistics

Unsupervised principal component analysis (PCA) and supervised partial least squares-discriminant analysis (PLS-DA) were applied to visualize, group, and classify the samples. Both statistical analyses were conducted in R software (version 3.4.1); PCA was performed using the R package stats and PLS-DA was carried out with the R package mixOmics. In addition, hierarchical cluster analysis (HCA) was applied to create heatmaps of the differentially expressed metabolites and to assign samples to clusters (R version 3.4.1, package heatmap).

### Univariate Statistics

Paired *t*-tests were performed to identify significant metabolite alterations in response to treatment (R version 3.4.1, package stats). To control type 1 error (i.e., false positives), q values were calculated using the Benjamini-Hochberg method (34). The level of significance was set at *p* ≤ 0.05 and *q* ≤ 0.2, respectively.

## Results

### A Clear Clustering Between Pre-treatment vs. Post-treatment 1 and Post-treatment 2

**Figure 2:**
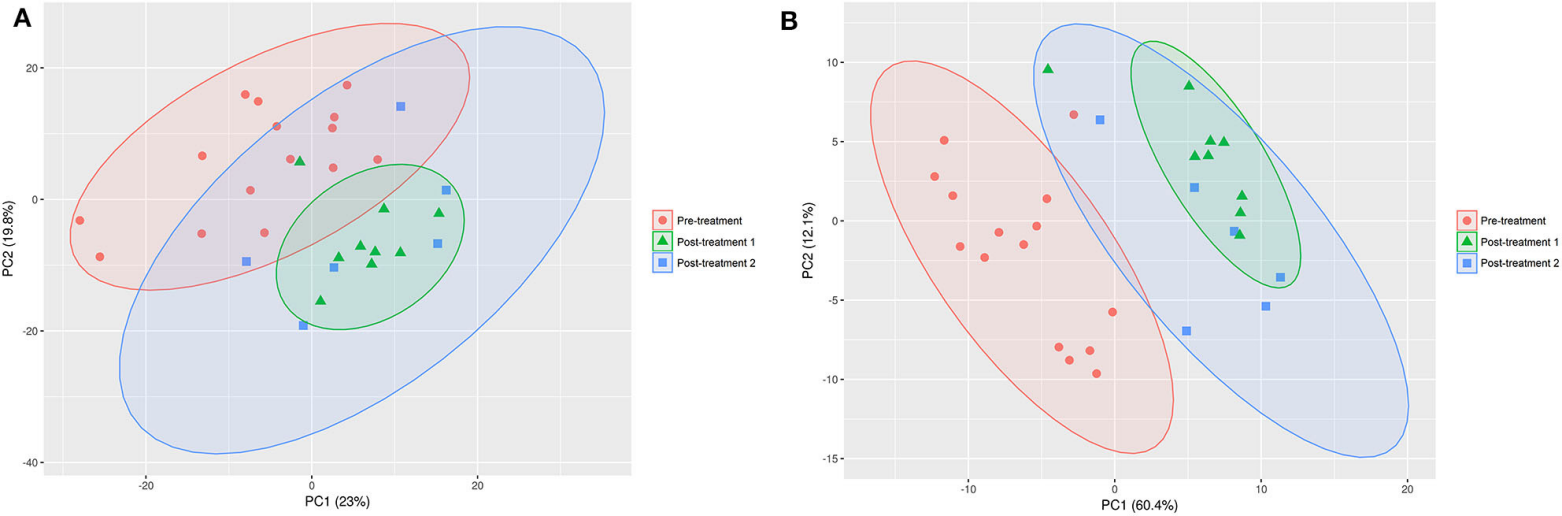
PCA **(A)** and PLS-DA **(B)** on the data set for pre- vs. post-treatment 1 and post-treatment 2. The confidence interval of the ellipses is 95% considering a normal distribution. Pre-treatment, *n* = 15, red circles; post-treatment 1, *n* = 9, green triangles; post-treatment 2, *n* = 6; blue squares.

The PCA (Figure 2A) and PLS-DA (Figure 2B) plots revealed a clustering for pre-treatment vs. post-treatment 1 (GAG+PRB) and post-treatment 2 (placebo), indicating that both of the treatments had a significant impact on the canine serum metabolome. In addition, despite the fact that the samples in the post-treatment 2 group are more distributed along principal component 2 in both PCA and PLS-DA, there was no clear separation between treatment 1 and treatment 2. This suggests that there was no difference between the two treatment groups on the serum metabolite levels overall.

### Various Serum Metabolites Changed Between Pre- and Post-treatment 1

**Figure 3:**
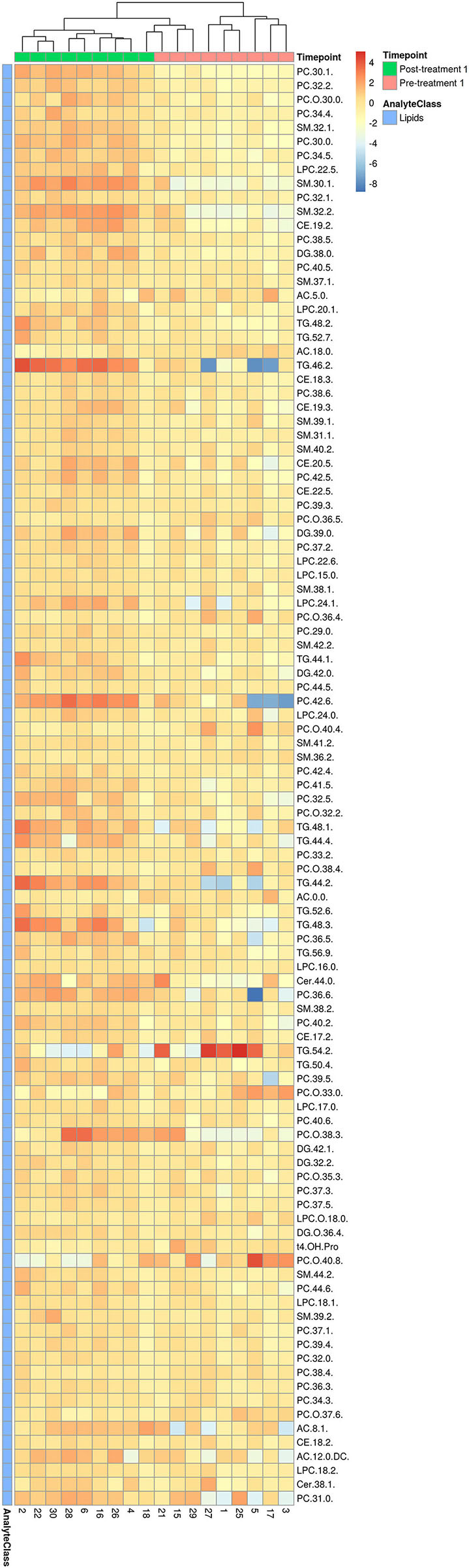
HCA for significantly changed metabolites between pre- and post-treatment 1. Hierarchical clustering and heatmap of all 103 metabolites that were identified to be significantly different (*p* ≤ 0.05 and *q* ≤ 0.2) in concentration between pre-treatment (*n* = 9) and post-treatment 1 (*n* = 9). Dog identification numbers are provided on the x-axis.

**Figure 4:**
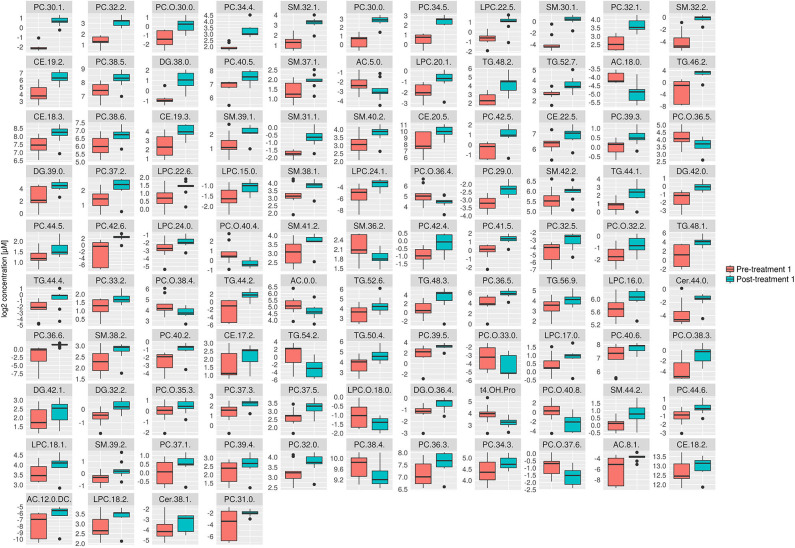
Box plots of all significantly changes metabolites between pre- and post-treatment 1. Box plots of significantly changed metabolites (*p* ≤ 0.05 and q ≤ 0.2) for the comparison of pre-treatment (*n* = 9) and post-treatment 1 (*n* = 9) are shown on the cleaned, imputed and log2-transformed data set. Concentration unit is μM. Boxplots show the median (bar), the interquartile range (box), whiskers (range) corresponding to maximal and minimal data, and suspected outliers (filled circles).

Consistent with the PCA and PLS-DA results (Figure 2), HCA based on significantly changed metabolites between pre- vs. post-treatment 1 showed a strong clustering between samples taken before and after treatment (Figure 3). Univariate statistical analysis showed that almost all significantly changed metabolites in response to treatment 1 were lipids, indicating an overall altered lipid metabolism after hydrolyzed diet therapy when combined with GAG+PRB administration (Figure 4). Glycerophospholipids, such as phosphatidylcholines (PCs), sphingolipids, such as sphingomyelins (SMs), as well as lysophosphatidylcholines (LPCs), and triglycerides (TGs) were the most affected metabolite classes. Specifically, multi-fold increases were seen after treatment 1 for PC(30:1) and PC(42:6), SM(30:1) and SM(32:2), LPC(22:5), and TG(46:2), TG(48:3), and TG(44:2).

### Various Serum Metabolites Changed Between Pre- and Post-treatment 2

**Figure 5:**
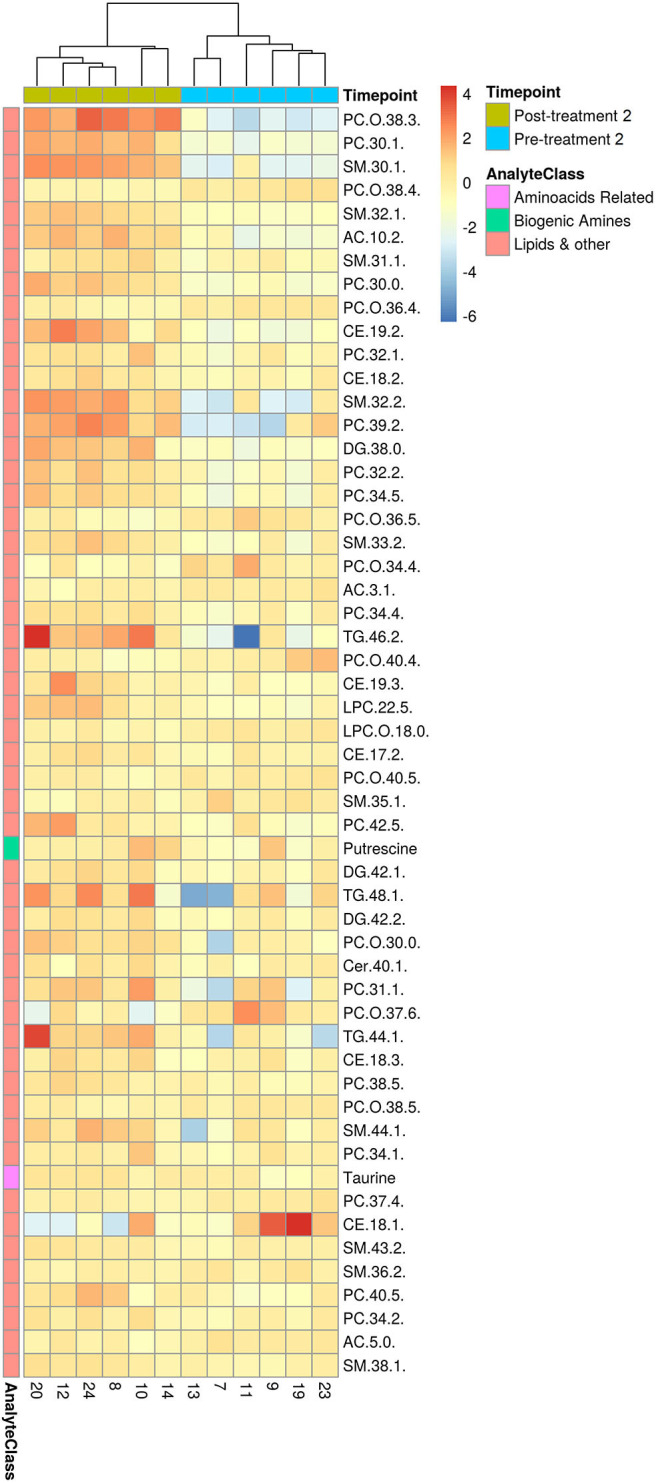
HCA for significantly changed metabolites between pre- and post-treatment 2. Hierarchical clustering and heatmap of all 54 metabolites that were identified to be significantly different (*p* ≤ 0.05 and *q* ≤ 0.2) in concentration between pre-treatment (*n* = 6) and post-treatment 1 (*n* = 6). Dog identification numbers are provided on the x-axis.

**Figure 6:**
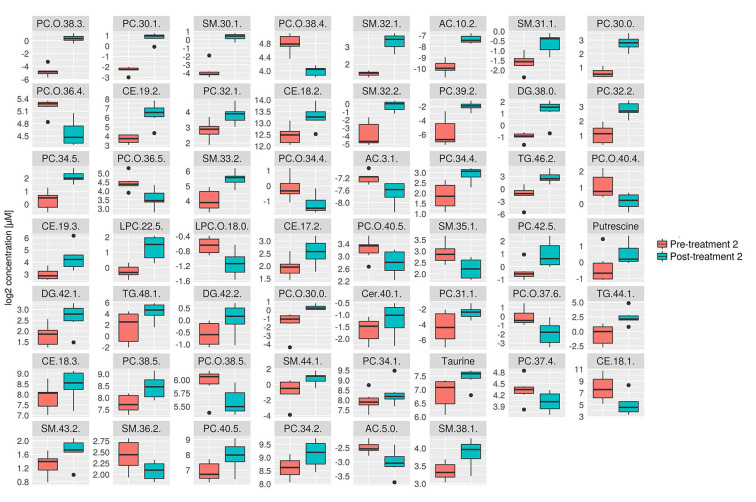
Box plots of all significantly changes metabolites between pre- and post-treatment 2. Box plots of significantly changed metabolites (*p* ≤ 0.05 and *q* ≤ 0.2) for the comparison of pre-treatment (*n* = 6) and post-treatment 2 (*n* = 6) are shown on the cleaned, imputed and log2-transformed data set. Concentration unit is μM. Boxplots show the median (bar), the interquartile range (box), whiskers (range) corresponding to maximal and minimal data, and suspected outliers (filled circles).

Consistent with the PCA and PLS-DA results (Figure 2), HCA based on significantly changed metabolites between pre- vs. post-treatment 2 showed a strong clustering between samples taken before and after treatment (Figure 5). Univariate statistical analysis showed that almost all significantly changed metabolites in response to treatment 2 were lipids, indicating an overall altered lipid metabolism (Figure 6), similar to the effects in response to treatment 1. Specifically, multi-fold increases were seen after treatment 2 for PC-O(38:3), PC(30:1), and PC(39:2), SM(30:1) and SM(32:2), DG(38:0), TG(44:1) and TG(46:2), CE(19:2) and AC(10:2). Notably, putrescine and taurine levels were increased ~1.5 times following treatment 2, which was different from the effects seen following treatment 1.

### Comparison of Serum Metabolomic Changes Between Treatment 1 and 2

**Figure 7:**
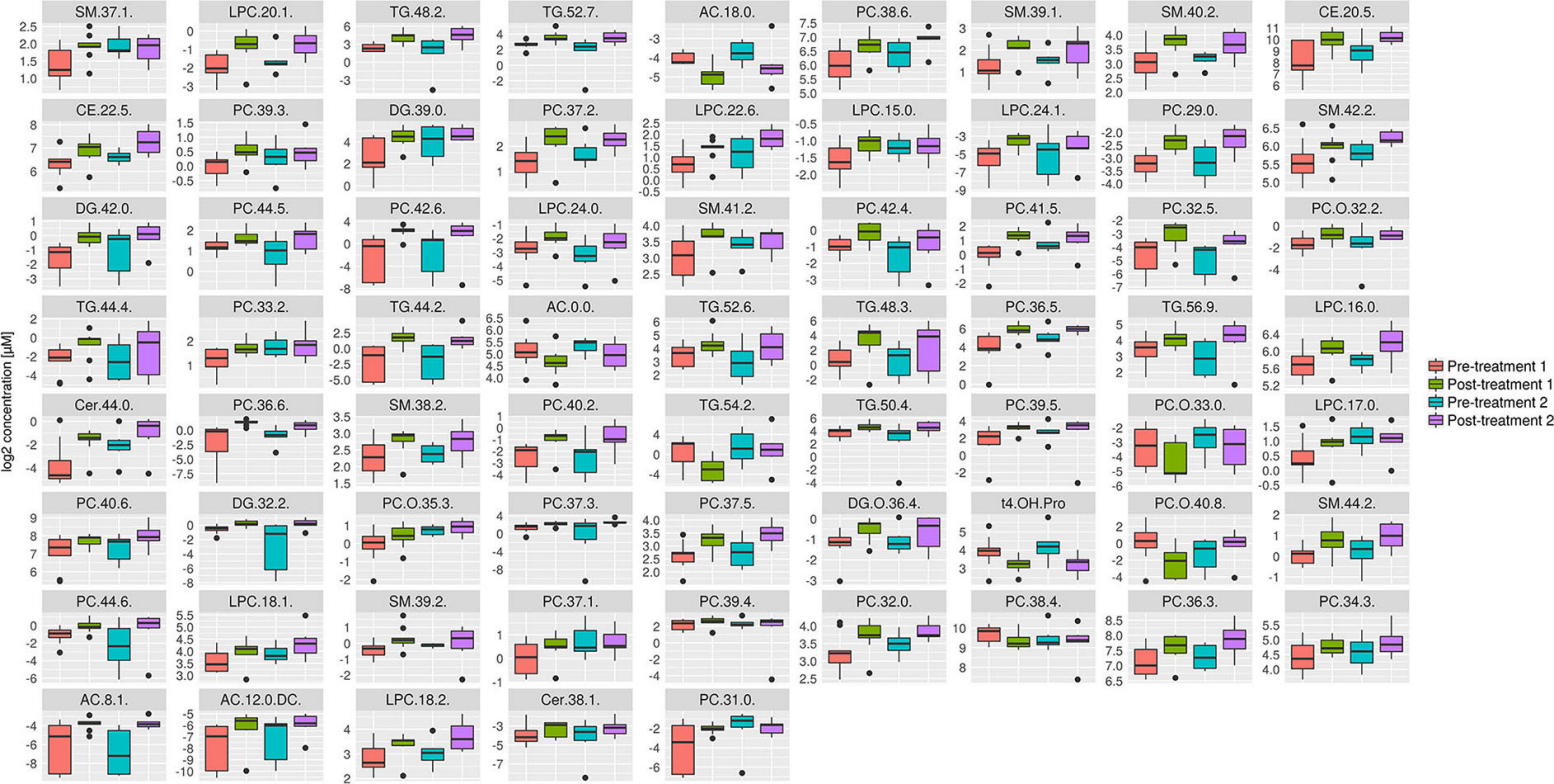
Box plots of all metabolites that were significantly changed in response to treatment 1, but not in response to treatment 2. Concentration unit is μM. Boxplots show the median (bar), the interquartile range (box), whiskers (range) corresponding to maximal and minimal data, and suspected outliers (filled circles).

**Figure 8:**
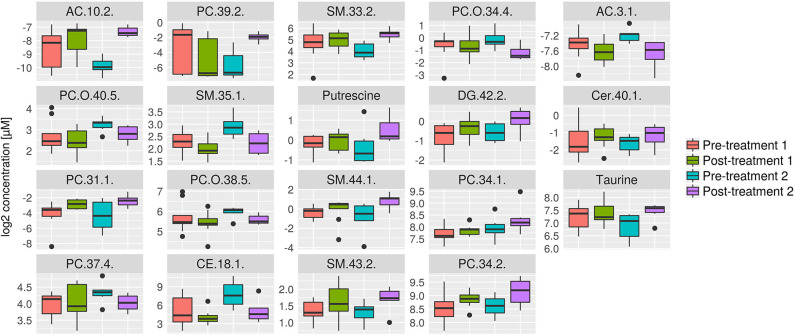
Box plots of all metabolites that were significantly changed in response to treatment 2, but not in response to treatment 1. Concentration unit is μM. Boxplots show the median (bar), the interquartile range (box), whiskers (range) corresponding to maximal and minimal data, and suspected outliers (filled circles).

Further analyses were performed to investigate which serum metabolites were uniquely changed by each treatment. The serum metabolites that were significantly changed after treatment 1 but not in response to treatment 2 are shown in Figure 7. Consistent with the univariate statistical analysis, significant changes were noted in lipids including PC(42:6), TG(44:2), and TG(48:3). The serum metabolites that were significantly changed following treatment 2 but not in response to treatment 1 are shown in Figure 8. Consistent with the univariate statistical analysis, lipids including PC(39:2) were increased significantly after in treatment 2 but not after treatment 1. Also, putrescine and taurine levels were increased significantly in treatment 2 group but not in treatment 1 group. In addition to single metabolite alterations, we also found that the sum of measured PCs was significantly increased in response to treatment 2, a result which was not found in response to treatment 1 (Figure S1).

## Discussion

In this study, a mass-spectrometric approach was applied to study metabolomic changes in serum samples from dogs with IBD in response to hydrolyzed diet with or without PRB and GAG supplementation. We found that the dogs' serum metabolome changed significantly after hydrolyzed diet, both with or without PRB and GAG supplement. Overall, lipids were greatly impacted by these treatments, with PCs and SMs showing the greatest increases in concentration. In addition, we found that simultaneous treatment with PRBs and GAGs in combination with the hydrolyzed diet increased more lipid metabolites compared to placebo therapy, which could indicate a possible beneficial effect of PRB and GAG supplementation in FRD.

The most significantly altered metabolites in response to treatment 1 (Purina HA and GAG+PRB) and treatment 2 (Purina HA and placebo) were PCs, which consist of a choline head group and a glycerophosphoric acid with a variety of saturated and unsaturated fatty acids (35). SMs, as an example of sphingolipids, were also significantly affected by treatment 1 and 2. They consist of sphingosine and a fatty acid, which mainly occur in cell membranes and are synthesized by the enzymatic transfer of a phosphocholine from phosphatidylcholine to ceramide (36). SM levels were increased after both treatment strategies, indicating that biochemical pathways in which these molecules are involved were impacted in IBD dogs. PCs and SMs have been shown to be essential structural components of intestinal membranes, providing integrity and protection to the intestinal mucosa (37, 38). Since the mucosal abnormalities in canine IBD include severe mucosal architectural changes (30, 31), the increase in PCs and SMs observed in this study may reflect a restoration of the mucosal barrier due to the protective effects of those glycerophospholipids on the intestinal cell integrity. Other metabolites found in membranes that were affected by both treatments were DGs and TGs, which also generally tended to be elevated after the treatments, suggesting improved lipid absorption as a potential sign of recovery of the intestinal mucosal integrity.

These findings are important because various studies have shown that the lipid metabolism could play a crucial role in modulating the homeostasis of the intestinal epithelium (39). Disruption of intestinal tight junctions, followed by an increase in intestinal permeability due to local inflammation or dysbiosis, could be a key trigger for IBD development (12). Maintaining lipid metabolism could serve as a protective mechanism for such a disruption (37). In particular, it has been shown that a type of sphingolipids, gangliosides, inhibits enterotoxin activity and thus contributes to maintaining the intestinal barrier integrity (40). This type of sphingolipids also inhibits adhesion and growth of *Escherichia coli* in infants with intestinal infections (41).

Amino acids and biogenic amines were hardly altered by the treatments, suggesting that the respective metabolic pathways involving these molecules were not changed by the hydrolyzed diet; only the levels of taurine and putrescine were increased following treatment 2. Taurine has various physiological functions, including a role in the development of central nervous, renal, and cardiovascular systems, stabilization of membranes, regulation of adipose tissue, and antioxidant effects (42–45). In addition, experiments in rats have shown that taurine can ameliorate the clinical signs of IBD (46). Animal models for IBD have also shown that dietary amino acids, such as tryptophan, could improve IBD as an adjunct to conventional therapy (45, 47, 48). High serum taurine levels could be considered as a biomarker for a positive outcome of IBD treatment and an adjunctive therapy for canine IBD. Putrescine, which is one of the biogenic amines, was also increased only following treatment 2 in our study. Interestingly, this metabolite was also found to be significantly increased in human IBD patients compared to healthy controls (49). Polyamines, including putrescine, determine the physiologic growth of intestinal mucosa, and elevated levels of polyamines seem to lead to the oxidative stress caused by the catabolism of polyamines (50). A sustained increase in putrescine amounts could therefore indicate persistent oxidative stress, and this oxidative stress could be involved in the pathogenesis of canine IBD (51) as well as human IBD (52). This indicates that treatment 2 (i.e., diet therapy without PRBs and GAGs) might not be entirely protective in its effect on gut homeostasis.

Contrary to genomic and proteomic studies, metabolomic analysis allows the evaluation of the simplest low molecular weight metabolites that are involved in disease processes (14, 53). The present study showed a positive effect of IBD treatment using PRBs and GAGs in addition to the hydrolyzed diet alone. Mechanistic effects of glycerophospholipids or TGs could be tested in our recently established intestinal cell models to better recapitulate canine intestine (54, 55).

The very first metabolomic study on canine IBD showed no significant alterations in lipid metabolism between healthy and IBD dogs (27). Interestingly, the authors discussed that the method they utilized, gas chromatography coupled with time-of-flight mass spectrometry, might have been a less sensitive method for lipid detection. In our study, we used UHPLC to detect even low levels of lipid metabolites in serum samples from IBD dogs. Another recently published study sought to overcome this weakness utilizing a hydrophilic interaction liquid chromatography of polar lipid classes (28). Interestingly, this study showed significant alterations in lipid metabolites before and after diet and glucocorticoid treatment in IBD dogs (28). However, this study only assessed phospholipids, while our current study examined a large number of phospholipids as well as a large number of other lipids and low molecular weight molecules. We used calibration curves and internal standards to quantify the amino acids and biogenic amines and a single-point internal standard calibration to quantify the lipids. These methods are considered to be more robust approaches compared to methods based on relative response factors of lipid analytes without internal standards. It is important to note that the study performed by Kalenyak et al. (28) employed a special novel protein diet (i.e., codfish), which is not commercially available in dogs. The results are therefore difficult to apply to a population of dogs treated with commercially available hydrolyzed diets, as was done in our study. In addition, their study only treated food-responsive IBD dogs with 4-week diet therapy, while our study showed stable response over a 10-week period, similar to the longer duration of treatment recommended in human celiac disease (56). Furthermore, Kalenyak et al., pointed out in their work that the samples were stored at −20°C for ~10 years, which was previously shown to have an impact on the serum metabolome (57). However, evidence of similar findings in lipid metabolome alterations at the time of clinical remission suggests that further investigations on lipidomics in canine IBD are needed. Studies that include such sensitive lipid metabolite analysis in larger samples from healthy and IBD dogs will likely elucidate biomarkers for early disease detection and help elucidate underlying pathogenic mechanisms.

One of the limitations of our study was the small sample size of animals used in this study. Therefore, it is possible that we may have overlooked some minor differences in metabolite profiles between treatment groups. However, our study showed moderate changes in lipid metabolite in IBD dogs after medical interventions that correlated with clinical improvements (clinical remission data will be published elsewhere). This suggests that a change in lipid metabolism is partially involved in the pathogenesis of canine IBD, similar to data in human IBD and mouse models of IBD (22–24). Fecal microbiome analysis was not performed in this study which, however, would have been interesting and may have linked some of the changes in the serum metabolome to possible dysbiosis in the microbiome. Further limitations of our study were that the dogs were client-owned and therefore fed individually by them, and that we have not included healthy controls to assess disease-specific changes in metabolomic perturbations.

In conclusion, our metabolomic study showed a significant increase in metabolites that protect gut membrane integrity after treatment with hydrolyzed diet in IBD dogs. Concomitant administration of PRBs and GAGs with a hydrolyzed diet of more than 70 days was well-tolerated by IBD dogs and resulted in additional improvements in selected serum lipid metabolites, possibly indicating an improvement of intestinal membrane integrity.

## Data Availability Statement

The original contributions presented in the study are included in the article/Supplementary Material, further inquiries can be directed to the corresponding authors.

## Ethics Statement

The animal study was reviewed and approved by the Animal Welfare and Ethical Review Board (AWERB) of the Royal Veterinary College (P302A3B70) the UK Home Office under The Animals (Scientific Procedures) Act 1986 (PPL 70/7393). Written informed consent was obtained from the owners for the participation of their animals in this study.

## Author Contributions

KA conceived of the idea for the research. KA, BG, and OG performed the clinical trial. SN, UM, MA, Y-JS, and JM performed the statistical analyses. YA, JM, and KA interpreted the data and reviewed literature. YA drafted and revised the manuscript. VD drafted the materials and methods section for the mass-spectrometry. DBorc, SS, BG, DBort, OG, TA, AW, JM, AJ, SN, and KA reviewed and edited the manuscript. MA performed statistical analysis and contributed figures to the manuscript. All authors read and approved the final manuscript.

## Conflict of Interest

SS is employed by the company Bioiberica SAU, Spain. KA does scientific consultancy for Bioiberica SAU. The authors declare that this study received funding from Bioiberica SAU. The funder had the following involvement with the study; providing the compounds used in the study. AJ, JM, and KA are founders of LifEngine Animal Health Laboratories, Inc., Rochester, MN, and co-founders of 3D Health Solutions, Inc., Ames, IA. The terms of this arrangement have been reviewed and approved by Iowa State University in accordance with its conflict of interest policies. The remaining authors declare that the research was conducted in the absence of any commercial or financial relationships that could be construed as a potential conflict of interest.
