# Supplementary material for: Treatment With Hydrolyzed Diet Supplemented With Prebiotics and Glycosaminoglycans Alters Lipid Metabolism in Canine Inflammatory Bowel Disease
Source: Front Vet Sci. 2020 Jul 30;7:451. doi: 10.3389/fvets.2020.00451 (PMC7406657; doi:10.3389/fvets.2020.00451)
Supplement: Supplementary file 1 [file Data_Sheet_1.PDF]

Supplementary Material

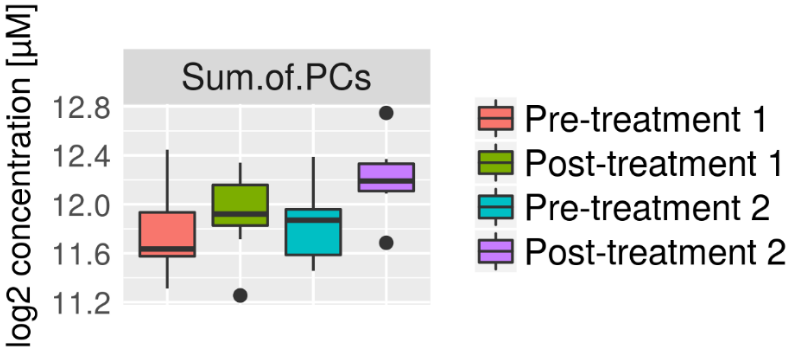

Figure S1. Sum of phosphatidylcholines was increased in response to treatment 2, but not in response to treatment 1.

Box plots of the sum of measured phosphatidylcholines (PC(25:0), PC(29:0), PC(30:0), PC(30:1), PC(31:0), PC(31:1), PC(32:0), PC(32:1), PC(32:2), PC(32:5), PC(32:6), PC(33:0), PC(33:1), PC(33:2), PC(33:4), PC(34:1), PC(34:2), PC(34:3), PC(34:4), PC(34:5), PC(35:1), PC(35:2), PC(35:3), PC(35:4), PC(35:5), PC(36:1), PC(36:2), PC(36:3), PC(36:4), PC(36:5), PC(36:6), PC(37:1), PC(37:2), PC(37:3), PC(37:4), PC(37:5), PC(37:6), PC(38:4), PC(38:5), PC(38:6), PC(38:7), PC(39:2), PC(39:3), PC(39:4), PC(39:5), PC(39:6), PC(39:7), PC(40:1), PC(40:2), PC(40:5), PC(40:6), PC(40:7), PC(40:8), PC(40:9), PC(41:3), PC(41:4), PC(41:5), PC(41:8), PC(42:4), PC(42:5), PC(42:6), PC(43:6), PC(44:5), PC(44:6), PC(44:10), PC(44:12), PC-O(30:0), PC-O(30:2), PC-O(32:0), PC-O(32:1), PC-O(32:2), PC-O(32:3), PC-O(33:0), PC-O(34:0), PC-O(34:1), PC-O(34:2), PC-O(34:3), PC-O(34:4), PC-O(35:3), PC-O(36:1), PC-O(36:2), PC-O(36:3), PC-O(36:4), PC-O(36:5), PC-O(36:6), PC-O(37:6), PC-O(38:3), PC-O(38:4), PC-O(38:5), PC-O(38:6), PC-O(40:4), PC-O(40:5), PC-O(40:6), PC-O(40:7), PC-O(40:8), PC-O(42:5), PC-O(42:6)) for the comparison of pre-treatment 1 (n=9) versus and post-treatment 1 (n=9) and pre-treatment 2 (n=6) versus post-treatment 2 (n=6). Concentration unit is µM. Boxplots show the median (bar), the interquartile range (box), whiskers (range) corresponding to maximal and minimal data, and suspected outliers (filled circles).

Table S1: Guaranteed analysis of Purina® Pro Plan® Veterinary Diets HA Hydrolyzed™ Canine Formula.

Crude Protein (Min)	18.0%
Crude Fat (Min)	9.5%

Crude Fiber (Max)	4.0%
Moisture (Max)	11.0%
Ash (Max)	7.0%
Calcium (Ca) (Min)	0.7%
Phosphorous (P) (Min)	0.6%

Table S2: Metabolites included in the statistical analysis. A total of 284 metabolites were included in this study.

**Metabolite class**	**Metabolite short name**	**Metabolite full name**
Acylcarnitines	AC(0:0)	Carnitine
Acylcarnitines	AC(2:0)	Acetylcarnitine
Acylcarnitines	AC(3:0)	Propionylcarnitine
Acylcarnitines	AC(3:1)	Propenoylcarnitine
Acylcarnitines	AC(5:0)	Valerylcarnitine
Acylcarnitines	AC(5:0-DC)	Glutarylcarnitine
Acylcarnitines	AC(5:1)	Tiglylcarnitine
Acylcarnitines	AC(6:0)	Hexanoylcarnitine
Acylcarnitines	AC(7:0)	Heptanoylcarnitine
Acylcarnitines	AC(7:0-DC)	Pimelylcarnitine
Acylcarnitines	AC(8:0)	Octanoylcarnitine
Acylcarnitines	AC(8:1)	Octenoylcarnitine

Acylcarnitines	AC(10:0)	Decanoylcarnitine
Acylcarnitines	AC(10:2)	Decadienoylcarnitine
Acylcarnitines	AC(12:0)	Dodecanoylcarnitine
Acylcarnitines	AC(12:0-DC)	Dodecanedioylcarnitine
Acylcarnitines	AC(12:1)	Dodecenoylcarnitine
Acylcarnitines	AC(13:0)	Tridecanoylcarnitine
Acylcarnitines	AC(14:0)	Tetradecanoylcarnitine
Acylcarnitines	AC(14:0-OH)	Hydroxymyristoylcarnitine
Acylcarnitines	AC(14:1)	Tetradecenoylcarnitine
Acylcarnitines	AC(14:2-OH)	Hydroxytetradecadienoylcarnitine
Acylcarnitines	AC(16:0)	Hexadecanoylcarnitine
Acylcarnitines	AC(16:1)	Hexadecenoylcarnitine
Acylcarnitines	AC(16:2)	Hexadecadienoylcarnitine
Acylcarnitines	AC(18:0)	Octadecanoylcarnitine
Acylcarnitines	AC(18:1)	Octadecenoylcarnitine
Acylcarnitines	AC(18:1-OH)	Hydroxyoctadecenoylcarnitine
Acylcarnitines	AC(18:2)	Octadecadienoylcarnitine
Acylcarnitines	AC(19:0)	Nonadecanoylcarnitine
Amino acids	Ala	Alanine
Amino acids	Arg	Arginine
Amino acids	Asn	Asparagine

Amino acids	Asp	Aspartic acid
Amino acids	Cit	Citrulline
Amino acids	Gln	Glutamine
Amino acids	Glu	Glutamic acid
Amino acids	Gly	Glycine
Amino acids	His	Histidine
Amino acids	Ile	Isoleucine
Amino acids	Lys	Lysine
Amino acids	Met	Methionine
Amino acids	Orn	Ornithine
Amino acids	Phe	Phenylalanine
Amino acids	Pro	Proline
Amino acids	Ser	Serine
Amino acids	Thr	Threonine
Amino acids	Trp	Tryptophan
Amino acids	Tyr	Tyrosine
Amino acids	Val	Valine
Amino acids	xLeu	Leucine + Isoleucine
Biogenic amines	ADMA	Asymmetric dimethylarginine
Biogenic amines	alpha-AAA	alpha-Aminoadipic acid
Biogenic amines	Carnosine	Carnosine

Biogenic amines	Creatinine	Creatinine
Biogenic amines	Kynurenine	Kynurenine
Biogenic amines	Met-SO	Methionine sulfoxide
Biogenic amines	Putrescine	Putrescine
Biogenic amines	Sarcosine	Sarcosine
Biogenic amines	SDMA	Symmetric dimethylarginine
Biogenic amines	Serotonin	Serotonin
Biogenic amines	Spermidine	Spermidine
Biogenic amines	t4-OH-Pro	trans-4-Hydroxyproline
Biogenic amines	Taurine	Taurine
Ceramides	Cer(34:1)	Ceramide 34:1
Ceramides	Cer(38:1)	Ceramide 38:1
Ceramides	Cer(40:1)	Ceramide 40:1
Ceramides	Cer(41:1)	Ceramide 41:1
Ceramides	Cer(42:1)	Ceramide 42:1
Ceramides	Cer(42:2)	Ceramide 42:2
Ceramides	Cer(43:1)	Ceramide 43:1
Ceramides	Cer(44:0)	Ceramide 44:0
Cholesteryl Esters	CE(16:0)	Cholesteryl ester 16:0
Cholesteryl Esters	CE(16:1)	Cholesteryl ester 16:1
Cholesteryl Esters	CE(17:0)	Cholesteryl ester 17:0

Cholesteryl Esters	CE(17:1)	Cholesteryl ester 17:1
Cholesteryl Esters	CE(17:2)	Cholesteryl ester 17:2
Cholesteryl Esters	CE(18:1)	Cholesteryl ester 18:1
Cholesteryl Esters	CE(18:2)	Cholesteryl ester 18:2
Cholesteryl Esters	CE(18:3)	Cholesteryl ester 18:3
Cholesteryl Esters	CE(19:2)	Cholesteryl ester 19:2
Cholesteryl Esters	CE(19:3)	Cholesteryl ester 19:3
Cholesteryl Esters	CE(20:4)	Cholesteryl ester 20:4
Cholesteryl Esters	CE(20:5)	Cholesteryl ester 20:5
Cholesteryl Esters	CE(22:5)	Cholesteryl ester 22:5
Cholesteryl Esters	CE(22:6)	Cholesteryl ester 22:6
Diglycerides	DG(32:1)	Diacylglycerol 32:1
Diglycerides	DG(32:2)	Diacylglycerol 32:2
Diglycerides	DG(34:1)	Diacylglycerol 34:1
Diglycerides	DG(34:3)	Diacylglycerol 34:3
Diglycerides	DG(36:2)	Diacylglycerol 36:2
Diglycerides	DG(36:3)	Diacylglycerol 36:3
Diglycerides	DG(36:4)	Diacylglycerol 36:4
Diglycerides	DG(38:0)	Diacylglycerol 38:0
Diglycerides	DG(38:5)	Diacylglycerol 38:5
Diglycerides	DG(39:0)	Diacylglycerol 39:0

Diglycerides	DG(41:1)	Diacylglycerol 41:1
Diglycerides	DG(42:0)	Diacylglycerol 42:0
Diglycerides	DG(42:1)	Diacylglycerol 42:1
Diglycerides	DG(42:2)	Diacylglycerol 42:2
Diglycerides	DG(44:3)	Diacylglycerol 44:3
Diglycerides	DG-O(34:1)	Diacylglycerol O-36:4
Diglycerides	DG-O(36:4)	Diacylglycerol O-34:2
Lysophosphatidylcholines	LPC(15:0)	Lysophosphatidylcholine 15:0
Lysophosphatidylcholines	LPC(16:0)	Lysophosphatidylcholine 16:0
Lysophosphatidylcholines	LPC(16:1)	Lysophosphatidylcholine 16:1
Lysophosphatidylcholines	LPC(17:0)	Lysophosphatidylcholine 17:0
Lysophosphatidylcholines	LPC(17:1)	Lysophosphatidylcholine 17:1
Lysophosphatidylcholines	LPC(18:0)	Lysophosphatidylcholine 18:0
Lysophosphatidylcholines	LPC(18:1)	Lysophosphatidylcholine 18:1
Lysophosphatidylcholines	LPC(18:2)	Lysophosphatidylcholine 18:2
Lysophosphatidylcholines	LPC(20:1)	Lysophosphatidylcholine 20:1
Lysophosphatidylcholines	LPC(20:4)	Lysophosphatidylcholine 20:4
Lysophosphatidylcholines	LPC(22:5)	Lysophosphatidylcholine 22:5
Lysophosphatidylcholines	LPC(22:6)	Lysophosphatidylcholine 22:6
Lysophosphatidylcholines	LPC(24:0)	Lysophosphatidylcholine 24:0
Lysophosphatidylcholines	LPC(24:1)	Lysophosphatidylcholine 24:1

Lysophosphatidylcholines	LPC-O(16:1)	Lysophosphatidylcholine O-16:1
Lysophosphatidylcholines	LPC-O(18:0)	Lysophosphatidylcholine O-18:0
Lysophosphatidylcholines	LPC-O(18:1)	Lysophosphatidylcholine O-18:1
Lysophosphatidylcholines	LPC-O(18:2)	Lysophosphatidylcholine O-18:2
Monosaccharides	H1	Hexose
Phosphatidylcholines	PC(25:0)	Phosphatidylcholine 25:0
Phosphatidylcholines	PC(29:0)	Phosphatidylcholine 29:0
Phosphatidylcholines	PC(30:0)	Phosphatidylcholine 30:0
Phosphatidylcholines	PC(30:1)	Phosphatidylcholine 30:1
Phosphatidylcholines	PC(31:0)	Phosphatidylcholine 31:0
Phosphatidylcholines	PC(31:1)	Phosphatidylcholine 31:1
Phosphatidylcholines	PC(32:0)	Phosphatidylcholine 32:0
Phosphatidylcholines	PC(32:1)	Phosphatidylcholine 32:1
Phosphatidylcholines	PC(32:2)	Phosphatidylcholine 32:2
Phosphatidylcholines	PC(32:5)	Phosphatidylcholine 32:5
Phosphatidylcholines	PC(32:6)	Phosphatidylcholine 32:6
Phosphatidylcholines	PC(33:0)	Phosphatidylcholine 33:0
Phosphatidylcholines	PC(33:1)	Phosphatidylcholine 33:1
Phosphatidylcholines	PC(33:2)	Phosphatidylcholine 33:2
Phosphatidylcholines	PC(33:4)	Phosphatidylcholine 33:4
Phosphatidylcholines	PC(34:1)	Phosphatidylcholine 34:1

Phosphatidylcholines	PC(34:2)	Phosphatidylcholine 34:2
Phosphatidylcholines	PC(34:3)	Phosphatidylcholine 34:3
Phosphatidylcholines	PC(34:4)	Phosphatidylcholine 34:4
Phosphatidylcholines	PC(34:5)	Phosphatidylcholine 34:5
Phosphatidylcholines	PC(35:1)	Phosphatidylcholine 35:1
Phosphatidylcholines	PC(35:2)	Phosphatidylcholine 35:2
Phosphatidylcholines	PC(35:3)	Phosphatidylcholine 35:3
Phosphatidylcholines	PC(35:4)	Phosphatidylcholine 35:4
Phosphatidylcholines	PC(35:5)	Phosphatidylcholine 35:5
Phosphatidylcholines	PC(36:1)	Phosphatidylcholine 36:1
Phosphatidylcholines	PC(36:2)	Phosphatidylcholine 36:2
Phosphatidylcholines	PC(36:3)	Phosphatidylcholine 36:3
Phosphatidylcholines	PC(36:4)	Phosphatidylcholine 36:4
Phosphatidylcholines	PC(36:5)	Phosphatidylcholine 36:5
Phosphatidylcholines	PC(36:6)	Phosphatidylcholine 36:6
Phosphatidylcholines	PC(37:1)	Phosphatidylcholine 37:1
Phosphatidylcholines	PC(37:2)	Phosphatidylcholine 37:2
Phosphatidylcholines	PC(37:3)	Phosphatidylcholine 37:3
Phosphatidylcholines	PC(37:4)	Phosphatidylcholine 37:4
Phosphatidylcholines	PC(37:5)	Phosphatidylcholine 37:5
Phosphatidylcholines	PC(37:6)	Phosphatidylcholine 37:6

Phosphatidylcholines	PC(38:4)	Phosphatidylcholine 38:4
Phosphatidylcholines	PC(38:5)	Phosphatidylcholine 38:5
Phosphatidylcholines	PC(38:6)	Phosphatidylcholine 38:6
Phosphatidylcholines	PC(38:7)	Phosphatidylcholine 38:7
Phosphatidylcholines	PC(39:2)	Phosphatidylcholine 39:2
Phosphatidylcholines	PC(39:3)	Phosphatidylcholine 39:3
Phosphatidylcholines	PC(39:4)	Phosphatidylcholine 39:4
Phosphatidylcholines	PC(39:5)	Phosphatidylcholine 39:5
Phosphatidylcholines	PC(39:6)	Phosphatidylcholine 39:6
Phosphatidylcholines	PC(39:7)	Phosphatidylcholine 39:7
Phosphatidylcholines	PC(40:1)	Phosphatidylcholine 40:1
Phosphatidylcholines	PC(40:2)	Phosphatidylcholine 40:2
Phosphatidylcholines	PC(40:5)	Phosphatidylcholine 40:5
Phosphatidylcholines	PC(40:6)	Phosphatidylcholine 40:6
Phosphatidylcholines	PC(40:7)	Phosphatidylcholine 40:7
Phosphatidylcholines	PC(40:8)	Phosphatidylcholine 40:8
Phosphatidylcholines	PC(40:9)	Phosphatidylcholine 40:9
Phosphatidylcholines	PC(41:3)	Phosphatidylcholine 41:3
Phosphatidylcholines	PC(41:4)	Phosphatidylcholine 41:4
Phosphatidylcholines	PC(41:5)	Phosphatidylcholine 41:5
Phosphatidylcholines	PC(41:8)	Phosphatidylcholine 41:8

Phosphatidylcholines	PC(42:4)	Phosphatidylcholine 42:4
Phosphatidylcholines	PC(42:5)	Phosphatidylcholine 42:5
Phosphatidylcholines	PC(42:6)	Phosphatidylcholine 42:6
Phosphatidylcholines	PC(43:6)	Phosphatidylcholine 43:6
Phosphatidylcholines	PC(44:5)	Phosphatidylcholine 44:5
Phosphatidylcholines	PC(44:6)	Phosphatidylcholine 44:6
Phosphatidylcholines	PC(44:10)	Phosphatidylcholine 44:10
Phosphatidylcholines	PC(44:12)	Phosphatidylcholine 44:12
Phosphatidylcholines	PC-O(30:0)	Phosphatidylcholine O-30:0
Phosphatidylcholines	PC-O(30:2)	Phosphatidylcholine O-30:2
Phosphatidylcholines	PC-O(32:0)	Phosphatidylcholine O-32:0
Phosphatidylcholines	PC-O(32:1)	Phosphatidylcholine O-32:1
Phosphatidylcholines	PC-O(32:2)	Phosphatidylcholine O-32:2
Phosphatidylcholines	PC-O(32:3)	Phosphatidylcholine O-32:3
Phosphatidylcholines	PC-O(33:0)	Phosphatidylcholine O-33:0
Phosphatidylcholines	PC-O(34:0)	Phosphatidylcholine O-34:0
Phosphatidylcholines	PC-O(34:1)	Phosphatidylcholine O-34:1
Phosphatidylcholines	PC-O(34:2)	Phosphatidylcholine O-34:2
Phosphatidylcholines	PC-O(34:3)	Phosphatidylcholine O-34:3
Phosphatidylcholines	PC-O(34:4)	Phosphatidylcholine O-34:4
Phosphatidylcholines	PC-O(35:3)	Phosphatidylcholine O-35:3

Phosphatidylcholines	PC-O(36:1)	Phosphatidylcholine O-36:1
Phosphatidylcholines	PC-O(36:2)	Phosphatidylcholine O-36:2
Phosphatidylcholines	PC-O(36:3)	Phosphatidylcholine O-36:3
Phosphatidylcholines	PC-O(36:4)	Phosphatidylcholine O-36:4
Phosphatidylcholines	PC-O(36:5)	Phosphatidylcholine O-36:5
Phosphatidylcholines	PC-O(36:6)	Phosphatidylcholine O-36:6
Phosphatidylcholines	PC-O(37:6)	Phosphatidylcholine O-37:6
Phosphatidylcholines	PC-O(38:3)	Phosphatidylcholine O-38:3
Phosphatidylcholines	PC-O(38:4)	Phosphatidylcholine O-38:4
Phosphatidylcholines	PC-O(38:5)	Phosphatidylcholine O-38:5
Phosphatidylcholines	PC-O(38:6)	Phosphatidylcholine O-38:6
Phosphatidylcholines	PC-O(40:4)	Phosphatidylcholine O-40:4
Phosphatidylcholines	PC-O(40:5)	Phosphatidylcholine O-40:5
Phosphatidylcholines	PC-O(40:6)	Phosphatidylcholine O-40:6
Phosphatidylcholines	PC-O(40:7)	Phosphatidylcholine O-40:7
Phosphatidylcholines	PC-O(40:8)	Phosphatidylcholine O-40:8
Phosphatidylcholines	PC-O(42:5)	Phosphatidylcholine O-42:5
Phosphatidylcholines	PC-O(42:6)	Phosphatidylcholine O-42:6
Sphingomyelins	SM(30:1)	Sphingomyelin 30:1
Sphingomyelins	SM(31:1)	Sphingomyelin 31:1
Sphingomyelins	SM(32:1)	Sphingomyelin 32:1

Sphingomyelins	SM(32:2)	Sphingomyelin 32:2
Sphingomyelins	SM(33:1)	Sphingomyelin 33:1
Sphingomyelins	SM(33:2)	Sphingomyelin 33:2
Sphingomyelins	SM(34:1)	Sphingomyelin 34:1
Sphingomyelins	SM(34:2)	Sphingomyelin 34:2
Sphingomyelins	SM(35:1)	Sphingomyelin 35:1
Sphingomyelins	SM(36:1)	Sphingomyelin 36:1
Sphingomyelins	SM(36:2)	Sphingomyelin 36:2
Sphingomyelins	SM(37:1)	Sphingomyelin 37:1
Sphingomyelins	SM(38:1)	Sphingomyelin 38:1
Sphingomyelins	SM(38:2)	Sphingomyelin 38:2
Sphingomyelins	SM(39:1)	Sphingomyelin 39:1
Sphingomyelins	SM(39:2)	Sphingomyelin 39:2
Sphingomyelins	SM(40:1)	Sphingomyelin 40:1
Sphingomyelins	SM(40:2)	Sphingomyelin 40:2
Sphingomyelins	SM(41:1)	Sphingomyelin 41:1
Sphingomyelins	SM(41:2)	Sphingomyelin 41:2
Sphingomyelins	SM(42:1)	Sphingomyelin 42:1
Sphingomyelins	SM(42:2)	Sphingomyelin 42:2
Sphingomyelins	SM(42:3)	Sphingomyelin 42:3
Sphingomyelins	SM(43:1)	Sphingomyelin 43:1

Sphingomyelins	SM(43:2)	Sphingomyelin 43:2
Sphingomyelins	SM(44:1)	Sphingomyelin 44:1
Sphingomyelins	SM(44:2)	Sphingomyelin 44:2
Triglycerides	TG(44:1)	Triacylglycerol 44:1
Triglycerides	TG(44:2)	Triacylglycerol 44:2
Triglycerides	TG(44:4)	Triacylglycerol 44:4
Triglycerides	TG(46:2)	Triacylglycerol 46:2
Triglycerides	TG(48:1)	Triacylglycerol 48:1
Triglycerides	TG(48:2)	Triacylglycerol 48:2
Triglycerides	TG(48:3)	Triacylglycerol 48:3
Triglycerides	TG(49:2)	Triacylglycerol 49:2
Triglycerides	TG(50:1)	Triacylglycerol 50:1
Triglycerides	TG(50:2)	Triacylglycerol 50:2
Triglycerides	TG(50:3)	Triacylglycerol 50:3
Triglycerides	TG(50:4)	Triacylglycerol 50:4
Triglycerides	TG(51:2)	Triacylglycerol 51:2
Triglycerides	TG(51:3)	Triacylglycerol 51:3
Triglycerides	TG(51:4)	Triacylglycerol 51:4
Triglycerides	TG(52:2)	Triacylglycerol 52:2
Triglycerides	TG(52:3)	Triacylglycerol 52:3
Triglycerides	TG(52:4)	Triacylglycerol 52:4

Triglycerides	TG(52:5)	Triacylglycerol 52:5
Triglycerides	TG(52:6)	Triacylglycerol 52:6
Triglycerides	TG(52:7)	Triacylglycerol 52:7
Triglycerides	TG(53:3)	Triacylglycerol 53:3
Triglycerides	TG(53:4)	Triacylglycerol 53:4
Triglycerides	TG(53:5)	Triacylglycerol 53:5
Triglycerides	TG(53:6)	Triacylglycerol 53:6
Triglycerides	TG(54:2)	Triacylglycerol 54:2
Triglycerides	TG(54:3)	Triacylglycerol 54:3
Triglycerides	TG(54:4)	Triacylglycerol 54:4
Triglycerides	TG(54:5)	Triacylglycerol 54:5
Triglycerides	TG(54:6)	Triacylglycerol 54:6
Triglycerides	TG(54:7)	Triacylglycerol 54:7
Triglycerides	TG(55:6)	Triacylglycerol 55:6
Triglycerides	TG(55:7)	Triacylglycerol 55:7
Triglycerides	TG(55:8)	Triacylglycerol 55:8
Triglycerides	TG(56:6)	Triacylglycerol 56:6
Triglycerides	TG(56:7)	Triacylglycerol 56:7
Triglycerides	TG(56:8)	Triacylglycerol 56:8
Triglycerides	TG(56:9)	Triacylglycerol 56:9
